# Oligodontia in the Clinical Spectrum of Syndromes: A Systematic Review

**DOI:** 10.3390/dj11120279

**Published:** 2023-12-04

**Authors:** Natália Lopes Castilho, Kêmelly Karolliny Moreira Resende, Juliana Amorim dos Santos, Renato Assis Machado, Ricardo D. Coletta, Eliete Neves Silva Guerra, Ana Carolina Acevedo, Hercílio Martelli-Junior

**Affiliations:** 1 Health Science Postgraduate Program, State University of Montes Claros, Montes Claros 39400-000, Brazil; nlcastilho@hotmail.com; 2 Laboratory of Oral Histopathology, Oral Care Center for Inherited Diseases, Health Sciences Faculty, University of Brasilia, Brasilia 70040-010, Brazil; kemellyresende@hotmail.com (K.K.M.R.); elieteneves@unb.br (E.N.S.G.); acevpoppe@gmail.com (A.C.A.); 3 Laboratory of Oral Histopathology, Health Sciences Faculty, University of Brasilia, Brasilia 70040-010, Brazil; juliana@zamorim.com; 4 Department of Oral Diagnosis and Graduate Program in Oral Biology, School of Dentistry, University of Campinas, Piracicaba 13414-018, Brazil; renatoassismachado@yahoo.com.br (R.A.M.); coletta@fop.unicamp.br (R.D.C.); 5 Oral Medicine and Oral Pathology, School of Dentistry, State University of Montes Claros, Unimontes, Montes Claros 39400-000, Brazil

**Keywords:** tooth agenesis, oligodontia, syndrome, systematic review

## Abstract

The aim of this systematic review was to describe the clinical and genetic features of syndromes showing oligodontia as a sign. The review was performed according to the PRISMA 2020 checklist guidelines, and the search was conducted using PubMed, Scopus, Lilacs, Web of science, Livivo, and EMBASE and supplemented by a gray literature search on Google Scholar and ProQuest, applying key terms relevant to the research questions. The systematic review identified 47 types of syndromes in 83 studies, and the most common was hypohidrotic ectodermal dysplasia, which was reported in 24 patients in 22 studies. Other common syndromes that reported oligodontia included Axenfeld–Rieger syndrome, Witkop’s syndrome, Ellis–van Creveld syndrome, blepharocheilodontic syndrome, and oculofaciocardiodental syndrome. The X-linked mode of inheritance was the most reported (n = 13 studies), followed by the autosomal dominant (n = 13 studies). The review describes the main syndromes that may have oligodontia as a clinical sign and reinforces the need for orodental–facial examining for adequate diagnosis and treatment of the affected patients. Molecular analysis in order to better understand the occurrence of oligodontia is imperative.

## 1. Introduction

Tooth agenesis is defined as the absence of teeth from the normal series due to a failure to develop and encompasses hypodontia, oligodontia, and anodontia [1]. The absence of up to five teeth is classified as hypodontia, the congenital absence of six or more teeth is defined as oligodontia, and anodontia refers to the complete absence of all teeth from the normal series [1]. Tooth development is regulated by a series of signaling pathways, and genetic mutations in specific genes have been described as the causes of such defects [2,3]. Moreover, environmental factors such as trauma, infections, toxins, and dietary deficiencies have been implicated and could interact with the genetic factors as a complex and multifactorial disease [4,5].

The occurrence of oligodontia can be observed as an isolated trait (non-syndromic oligodontia) or accompanying other features as part of a syndrome [6,7,8,9]. In cases of non-syndromic oligodontia, the congenital absence of teeth stands as the sole discernible clinical characteristic [10]. These instances are relatively uncommon, with a prevalence that varies from 0.08% to 0.36%, contingent upon the specific population under study [11,12,13]. Nevertheless, the intricate interplay between oligodontia and syndromes has increasingly captured attention. The association of oligodontia with over 60 different syndromes, including but not limited to hypohidrotic ectodermal dysplasia (HED), Rieger’s syndrome, Down’s syndrome, and Van der Woude’s syndrome [14], underscores its significance as a phenotypic marker and a consequence of intricate genetic interactions. Notably, while oligodontia assumes a central role as a diagnostic hallmark for numerous syndromes, additional dental anomalies may co-occur. These anomalies encompass microdontia, shortened roots, dental impactions, delayed tooth formation, delayed eruption, canine and premolar transpositions, taurodontism, and enamel hypoplasia [2,15,16]. Tooth development is orchestrated by conserved signaling pathways that facilitate communication between ectodermal and mesenchymal tissues. In humans, mutations in *WNT10A* are the most commonly reported in the genetic etiology for syndromic oligodontia, and *PAX9* mutations are the most commonly reported genetic etiology for isolated oligodontia [17].

Patients with oligodontia present serious deficiencies in their quality of life due to decreased masticatory function, phonetic ability, and maxillofacial aesthetics [18,19]. As oligodontia can be a clinical manifestation of a large and heterogenous group of syndromes with multiple signs and symptoms, this systematic review aims to summarize the available literature concerning the presence of oligodontia in syndromes, emphasizing the phenotype and the molecular etiology in order to assist in the diagnosis and management of patients.

## 2. Materials and Methods

### 2.1. Protocol and Registration

This systematic review was performed according to the preferred reporting items for systematic reviews and meta-analyses (PRISMA) checklist [20], and its protocol was recorded in the International Prospective Register of Systematic Reviews (PROSPERO) database under registration number CRD42020190814.

### 2.2. Eligibility Criteria

The PICOS approach was used to formulate the question for this study: P—participants (syndromic patients with oligodontia); I—intervention (none), C—comparison (none); O—outcomes (frequency and types of syndromes associated with oligodontia, pattern of missing teeth, and frequency of pathogenic variants); S—study (case reports and case series).

The criteria for the exclusion of articles were as follows: (1) non-syndromic cases (n = 8); (2) studies that did not report (or reported unclear) dental X-rays (n = 80); (3) studies that did not report representative cases of oligodontia (n = 30); (4) studies that report cases of hypodontia or anodontia (n = 13); (5) studies that do not include pattern of tooth agenesis (n = 8); (6) reviews, letters, conference abstract, personal opinions, and in vitro or in vivo animal studies (n = 3); (7) full-text copy not available (n = 37); (8) articles that were not in Roman alphabet (n = 2); (9) studies with absence of clinical information (n = 23).

### 2.3. Study Selection

The selection process of the studies was performed using an individual search in each bibliographic database: PubMed, Scopus, Lilacs, Web of science, Livivo, and EMBASE. A gray literature search was conducted using Google Scholar and ProQuest. The research was performed in December 2022. However, a second literature search was performed using the same terms on 8th August 2021, retrieving articles published between January and August 2023. The search strategy can be assessed in Supplementary Table S1. The duplicate references were removed by reference manager software (EndNote X7, Thomson Reuters, Toronto, ON, Canada). All references were transferred and worked on the Rayyan (Rayyan, Qatar Computing Research Institute, Qatar Foundation, Doha, Qatar), developed specifically to expedite the initial screening of abstracts and titles [21].

The course of the research was established in two distinct phases. In the first phase, two authors (N.L.C. and K.K.M.R.) independently read all titles and abstracts, taking into account the eligibility criteria initially defined. In cases of no consensus, a third author (A.C.A.) was involved who determined which articles would be included in the second phase. The second phase was carried out by the same authors, who performed a full-text reading of the screened articles. Disagreements were solved via discussions involving the third author (A.C.A.).

### 2.4. Data Collection Process and Data Items

The data collection process was carried out by the two authors (N.L.C. and K.K.M.R.) who initially selected the articles in the two phases, in which the necessary and relevant information of each study was collected. The information was checked by a third author (A.C.A.).

### 2.5. Risk of Bias within Studies

The risk-of-bias was assessed by two authors (N.L.C. and K.K.M.R.) using the Joanna Briggs Institute Critical Appraisal Tools for Studies Reporting Prevalence Data for Use in Systematic Reviews—referred-to Case Reports [22]. The authors scored each item as “yes”, “no”, “unclear”, or “not applicable” when assessing the quality of each included study. Decisions about scoring were discussed by all reviewers. A study was characterized as having a high risk of bias when it reached a “yes” score of up to 49%, moderate when 50% to 69%, and low when >70%.

### 2.6. Interaction Analysis

The functional relevance of identified genes was further investigated with STRING, version 11.0 (a search tool for the retrieval of interacting genes, http://string-db.org, accessed on 1 November 2023), which provides a *p*-value after applying a false discovery rate for correction of multiple testing.

## 3. Results

**Figure 1:**
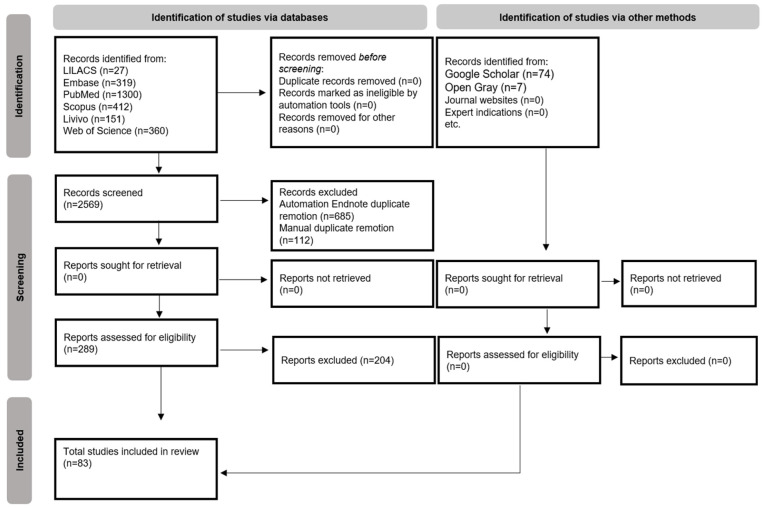
Flow diagram of literature search and selection criteria adapted via the preferred reporting items for systematic reviews and meta-analyses (PRISMA) [20].

The searches conducted in the six databases resulted in 2569 scientific articles. After removal of duplicates, 1772 articles were totalled. The gray literature search resulted in 81 articles. After reading the titles and abstracts in the first phase, 288 articles were selected for the next phase. At the end of reading the full articles (second phase), 83 articles were included for the qualitative synthesis [23,24,25,26,27,28,29,30,31,32,33,34,35,36,37,38,39,40,41,42,43,44,45,46,47,48,49,50,51,52,53,54,55,56,57,58,59,60,61,62,63,64,65,66,67,68,69,70,71,72,73,74,75,76,77,78,79,80,81,82,83,84,85,86,87,88,89,90,91,92,93,94,95,96,97,98,99,100,101,102,103,104,105]. The review process is schematized in a flowchart depicted on Figure 1.

The main data of each selected article are summarized in Table 1. Together, the studies reported 97 patients, ages ranging from 3 to 75 years old, with oligodontia as a clinical feature of different types of syndromes. The risk-of-bias assessment in each study is reported in Supplementary Table S2. All included articles were classified as having a low risk of bias, except for eight articles. These articles had unclear information regarding the demographics of the patients, the timeline of diagnosis, and, most importantly, the diagnostic tests used for the final diagnosis.

The syndromes more frequently identified, in decreasing order, were HED (24 patients in 22 studies), Axenfeld–Rieger syndrome (ARS) (9 patients in 5 studies), blepharocheilodontic syndrome (BCDS) (7 patients in 2 studies), Witkop’s syndrome (4 patients in 4 studies), oculofaciocardiodental syndrome (3 patients in 3 studies), incontinentia pigmenti (3 patients in 3 studies), Hallermann–Streiff syndrome (3 patients in 3 studies), polycistic ovarian syndrome (3 patients in 2 studies), Ellis–van Creveld syndrome (EVCS) (2 patients in 2 studies), Down’s syndrome (2 patients in 2 studies), Carvajal syndrome (2 patients in 2 studies), Carpenter syndrome (2 patients in 2 studies), and Kabuki syndrome (2 patients in 2 studies). Other uncommon syndromes listed in Table 1 were reported in one patient each.

Out of 97 patients, 10 patients were affected by oligodontia in both primary and permanent dentitions; in 2 cases, oligodontia was observed in the primary dentition; and in the other cases, oligodontia was observed in the permanent dentition (Table 1). The number of missing teeth ranged from 6 to 13 in the primary dentition and from 6 to 27 in the permanent dentition. In the deciduous dentition, the absence of the first molars and lateral and central incisors was observed in 76.9% of patients, and the second molars and canines were absent in 61.5% of patients (Table 1). In the permanent dentition, lateral incisors (76.2% of patients), first molars (75.5% of patients), central incisors (69.7% of patients), premolars (66% of patients), and second molars (40% of patients) were the more-affected teeth (Table 1).

The genetic profile of the different studies is summarized in Table 1. HED was the most-found syndrome, and the X-linked mode of inheritance was the most common for this syndrome. The most reported mode of inheritance for oculofaciocardiodental syndrome, incontinentia pigmenti and Christ–Siemens–Touraine syndrome was X-linked. The mode of inheritance for Carvajal syndrome, Noonan syndrome, ARS, Witkop’s syndrome, Apert syndrome, BCDS, and Kabuki syndrome was the autosomal dominant, whereas microcephalic osteodyplastic primordial dwarfism type II, tricho-odonto-onychodermal dysplasia, and EVCS were reported under autosomal recessive (Table 1).

**Figure 2:**
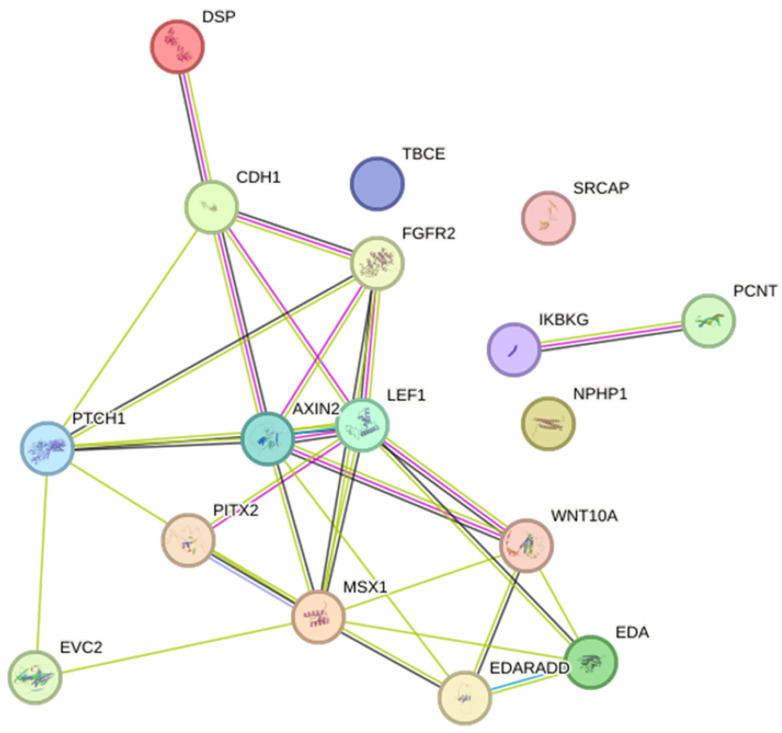
Protein–protein interaction network with the genes associated with syndromes with oligodontia. Two nodes of interactions, involving *AXIN2*, *CDH1*, *DSP*, *EDA*, *EDARADD*, *EVC2*, *FGFR2*, *LEF1*, *MSX1*, *PTCH1*, *PITX2*, and *WNT10A (p* < 1.0 × 10^−16^) and between *PCNT* and *IKBKG (p* = 0.04) were identified. Different colors represent different levels of evidence of connection between proteins; light blue represents curated databases, purple experimental evidence, green gene neighborhood, light green evidence from text mining, black co-expression, and violet protein homology. This analysis had an average confidence score of 0.657, suggesting a low rate for false-positive interactions.

Seven studies reported mutations in *EDA* or *WNT10A* in HED, Christ–Siemens–Touraine syndrome, tricho-odonto-onychodermal dysplasia, and odonto-onychodermal dysplasia. Others studies have reported mutations in *AXIN2* (HED), *CDH1* (blepharocheilodontic syndrome), *DSP* (Carvajal/Naxos syndrome), *EDARADD* (HED), *EVC2* (EVCS), *FGFR2* (Beare–Stevenson syndrome), *PCNT* (microcephalic osteofysplastic dwarfism type 2), *PITX2* (ARS), *PTCH1* (basal cell nevus syndrome), *IKBKG* (incontinentia pigmenti), *LEF1* (HED), *MSX1* (Witkop’s syndrome), *NPHP1* (juvenile nephronophtisis), *SRCAP* (Floating-Harbor syndrome), and *TBCE* (Sanjad–Sakati syndrome). Together, these genes participate in 80 biological processes and 19 pathways. The most significant biological processes were odontogenesis (GO:0042476; *p* = 1.56 × 10^−7^), gland development (GO:0048732; *p* = 7.29 × 10^−6^) and epithelium development (GO:0060429; *p* = 1.65 × 10^−5^), and the pathways were of pathways in cancer (hsa05200; *p* = 4.55 × 10^−5^), basal cell carcinoma (hsa05217; *p* = 4.55 × 10^−5^), and pathways of the gastric cancer (hsa05226; *p* = 4.55 × 10^−5^) (Supplementary Tables S3 and S4). The networks included 17 predicted interactions (Figure 2).

## 4. Discussion

Within the realm of syndromes characterized by oligodontia, questions arise regarding the consistency of this phenotype across cases, its varying expressiveness, its diagnostic utility, and the specific teeth most affected. This review sought to comprehensively address these queries by collating pertinent information from a diverse array of syndromes exhibiting oligodontia within their clinical spectrum. The exploration commenced by surveying the literature and transcending temporal constraints, leading to the identification of 47 distinct syndromes cataloged within the Online Mendelian Inheritance in Man. Among these, HED emerged as the most frequent. The hallmark trifecta of HED, involving hair, teeth, and sweat gland anomalies [106], was evident in the affected patients. The spectrum of dental agenesis in HED spans mainly oligodontia, but reports of hypodontia and even anodontia are found in the literature, with a predilection for the mandible. This remarkable variability necessitates close attention for correct diagnosis [107,108]. Notably, the distinctive conical shape of the anterior teeth, when present, offers a diagnostic clue. Furthermore, the potential confluence of maxillary retrusion, sagittal jaw underdevelopment, jaw displacement, and craniofacial alterations underlines the complex interplay of factors characterizing this syndrome [107,108,109].

Within our systematic review, several syndromes stood out for their prevalence and diverse oral manifestations. One of the noteworthy conditions identified was Ellis–van Creveld syndrome (EVCS), an autosomal recessive skeletal dysplasia. EVCS presents a range of oral phenotypes alongside limb abnormalities, including occlusion irregularities, labiogingival adhesions, hypertrophied labiogingival frenulum, accessory frenula, serrated incisal margins, dental transposition, diastemas, conical teeth, enamel hypoplasia, and congenital absence of multiple teeth [110,111]. The propensity for premature eruption or exfoliation further complicates the dental anomalies associated with EVCS. In our study, we observed an average of 6 deciduous teeth (one case) and 8.5 permanent teeth (two cases) missing in individuals with reported *EVC2* mutations. Another syndrome we examined was oculofaciocardiodental syndrome, a rare multi-systemic anomaly, with a higher prevalence in females. This syndrome showcases the intricate interplay between congenital cataracts, facial dysmorphisms, and dental anomalies, including radiculomegaly and oligodontia, in addition to congenital heart defects [77,112]. In our study, we noted an average of nine permanent teeth (three cases) missing in individuals with reported *WNT10A* mutations. Witkop syndrome, an uncommon autosomal dominant genetic disorder attributed to mutations in *MSX1*, held our attention due to its distinct dental and nail dysplasia characteristics. This syndrome is primarily marked by dental agenesis, primarily oligodontia, although the absence of up to five teeth (hypodontia) is also reported. Dental features often include conical-shaped teeth and teeth with narrow crowns [113]. In our study, we observed an average of 16.75 permanent teeth (four cases) missing in individuals with reported *MSX1* mutations. Axenfeld–Rieger syndrome (ARS), a condition characterized by ocular dysgenesis and systemic anomalies affecting the teeth, heart, craniofacial structure, and abdominal wall, was another significant focus. ARS is frequently associated with a 6p25 distal microdeletion but may also manifest in connection with other genetic loci such as 4q25 or 13q14. Several genes, including *FOXC1*, *FOXC2*, and *FKHL7*, have been implicated in the context of ARS [29,114]. In our study, we found an average of 9 primary teeth (one case) and 14.3 permanent teeth (nine cases) missing in individuals with reported *PITX2* mutations. Lastly, we explored branchio-oculofacial syndrome (BCDS), an autosomal dominant disorder known for congenital facial clefting, oligodontia, euryblepharon, lagophthalmos, and ectropion. While the extent of its expression can vary, the common features often encompass cleft lip and/or palate, ectropion, and lagophthalmos [76,115]. In our study, we observed an average of 8.5 primary teeth (four cases) and 15.4 permanent teeth (seven cases) missing in individuals with reported *CDH1* mutations.

Interrogating the consistency of oligodontia across syndromic cases is of paramount importance. The range of dental presentations, spanning from hypodontia to anodontia, underscores the variable expressiveness within these syndromes. Consequently, the manifestation of oligodontia should be viewed as a continuum instead of an absolute trait. This variable expressivity poses challenges in diagnosis and underscores the importance of considering broader phenotypic traits in conjunction with dental anomalies. Regarding the most-affected teeth, patterns emerged from the collated data. In deciduous dentition, absence of first molars and lateral and central incisors was observed in the majority of patients, with second molars and canines affected in a significant proportion. In permanent dentition, lateral incisors, first molars, central incisors, premolars, and second molars exhibited the highest susceptibility to oligodontia. HED is the most frequently mentioned syndrome with oligodontia, while *EDA* and *WNT10A* mutations constitute the most frequently determined genetic cause: 30.4% of the syndromic oligodontia. These findings are consistent with previous reports which established *WNT10A* variants accounting for up to 50% of various HED syndromes with missing teeth [17,116]. These patterns may offer clues for diagnosis and genetic assessment. However, as previously reported, there are several mechanisms that are involved in tooth development and other tissues of the body, establishing very heterogeneous phenotypes in affected individuals. Thus, radiographic and molecular diagnoses may be necessary. The analysis of dental radiographs is an important part of the diagnostic process in daily clinical practice, and interpretation by an expert includes teeth detection and numbering [117,118,119]. In the reading of the articles included, panoramic radiographs with poor quality were evidenced, which can affect the diagnosis and consequent interpretation of the case report. The detailed radiographic report of the observed alterations was also absent in most studies.

In some situations, in the differential diagnosis process, sequencing analysis is useful, and further exploration of the identified mutations can assist in the interpretation of the phenotypes (genotype–phenotype correlation). The important role of genetics has been increasingly recognized in recent years with regard to the understanding of dental anomalies such as tooth agenesis [120]. However, many of the included studies in this systematic review did not perform molecular analysis. Only 23 studies [41,43,52,53,56,63,66,67,68,70,73,74,76,78,79,87,88,89,90,92,94,98,105] performed genetic analysis and reported the genetic variants associated with the syndromes. These genes can be grouped into two major groups: one with crucial roles at multiple stages of tooth development, also involving skin and sweat glands (*AXIN2, CDH1, DSP, EDA, EDARADD, EVC2, FGFR2, LEF1, MSX1, PITX2,* and *WNT10A*), which are involved in the signal pathway essential for ectodermal structure development [121,122]; the other with genes that intermediate cellular function and development (*NPHP1, PCNT, PTCH1, IKBKG, SRCAP,* and *TBCE*). Interestingly, some of the identified genes are also associated with non-syndromic oligodontia, but in these cases, the mutations cause reduced expression, decreased receptor-binding affinity, or altered signaling-intensity of the mutated protein, whereas the mutations associated with syndromic oligodontia are characterized by a more intense impact on protein function [123].

The teeth affected by oligodontia often exhibit various dental anomalies, such as reduced size, a conical shape, and delayed eruption [12,121,122,123,124,125,126]. These shared features suggest the influence of similar genetic mechanisms [124]. Our findings align with previous research [120], indicating that permanent dentition is more frequently affected by oligodontia compared to primary dentition. In our study of 97 patients, we observed bilateral agenesis of maxillary lateral incisors in 64 patients, while 13 patients displayed unilateral absence of the second mandibular premolar. It is worth noting that clinical studies have reported that bilateral agenesis of the maxillary lateral incisors is more common than unilateral agenesis, and unilateral agenesis of the second mandibular premolar is more prevalent than bilateral cases [127].

However, it is important to acknowledge the limitations of this study. One significant limitation arises from the varied terminology used to define oligodontia, including terms such as severe hypodontia or partial anodontia. This inconsistency in nomenclature can lead to challenges in accurately classifying cases. Additionally, the absence of radiographic data for correct oligodontia diagnosis was a noteworthy limitation that impacted our sample size. Many studies included in this review did not perform genetic tests, which precluded a more comprehensive phenotype–genotype correlation analysis. Furthermore, some very rare disorders may not have been reported in the literature, potentially limiting the scope of our review with regard to syndromes associated with oligodontia.

In addition to its academic significance, the findings from this review hold substantial implications for clinical practice. Identifying specific dental anomalies and their association with syndromes can play a pivotal role in the diagnostic process. When assessing patients with oligodontia, it is essential for clinicians to consider the potential presence of an underlying syndrome. The number and type of missing or affected teeth can serve as valuable diagnostic clues, but a holistic evaluation of the patient’s overall clinical presentation, including physical and developmental features, is indispensable. Furthermore, the genetic insights garnered from this review offer the potential for more precise and personalized patient care. Understanding the genetic underpinnings of syndromic oligodontia can facilitate early diagnosis, inform genetic counseling, and guide treatment planning. Genetic testing, where applicable, can provide confirmation and assist in tailoring therapeutic strategies. This knowledge can also pave the way for the development of targeted therapies or interventions with which to address the dental and craniofacial challenges associated with syndromic oligodontia.

Looking ahead, there is a compelling need for further research in this field. Delving deeper into the genetic underpinnings of syndromic oligodontia and exploring the phenotypic heterogeneity in greater detail are paramount. Studies that employ consistent terminology and diagnostic methods will contribute to a more comprehensive understanding of this complex condition. Additionally, further investigations into genotype–phenotype correlations and the development of standardized diagnostic criteria for syndromic oligodontia are warranted. Comprehensive radiographic assessment and molecular analysis will be instrumental in elucidating the underlying mechanisms and genetic factors associated with these syndromes.

## 5. Conclusions

The identification of specific phenotypes associated with oligodontia can significantly reduce diagnostic uncertainty, particularly when these phenotypes are highly predictive of a specific syndrome. Based on the insights gained from this review, it is advisable that clinicians and geneticists, during the diagnosis process of patients with oligodontia, remain vigilant for the most common syndromes with oligodontia as a clinical sign. These syndromes include hypohidrotic ectodermal dysplasia (HED), Axenfeld–Rieger Syndrome (ARS), Witkop’s syndrome, Ellis–van Creveld syndrome (EVCS), Branchio-oculofacial syndrome (BCDS), and oculofaciocardiodental syndrome. These findings contribute to a more informed and precise diagnostic approach, ultimately enhancing patient care and management.

## Figures and Tables

**Table 1:** Manifestations of oligodontia in primary and permanent dentition in the included articles (n = 83).

Author Year/Country	Geographic Origin	Type of Study (CR/SC)	Syndrome Name (#OMIM)	Sex (M/F)	Age (Yrs)	Mode of Inheritance (AD/AR/X-LINKED)	Causative Gene	Dentition	Number of Teeth	Affected Teeth	Systemic Manifestations	Craniofacial Manifestations
Abdulla et al., 2019 [23]/Kingdom of Saudi Arabia	NR	CR	Hypohidrotic ectodermal dysplasia (#305100)	M	7	NR	NR	Permanent	14	12,14, 15, 22, 24, 25, 31, 32, 34, 35, 41, 42, 44, 45	No production of tears, sweat pores were poorly visible	Hair over the scalp was sparsely distributed, scanty eyebrows and eyelashes, hyperpigmentation around the eyes, depressed nasal bridge, prominent supraorbital ridge, everted dry lips, delayed eruption of permanent dentition, bone ridge on the mandibular anterior region was very thin, cone-shaped crowns, conical maxillary anterior teeth, oligodontia
Abs et al., 1994 [24]/Belgium	NR	CR	Wolff–Parkinson–White syndrome (#194200)	F	22	NR	NR	Permanent	11	14, 15, 17, 12, 22, 25, 27, 35, 37, 45, 47	Discrete cubitus valgus, hygonadotropic hypogonadism, ovaries had a prepubertal aspect without stigmata of ovulation, negative T-waves in leads 111 and AVF, low level of luteinizing hormone (LH), gonadotropinreleasing hormone (GnRH), folliclestimulating hormone (FSH).	Microdontia, retention of deciduous teeth, oligodontia
Aditya et al., 2012 [25]//India	NR	CR	Fahr’s syndrome (#213600)	F	23	NR	NR	Permanent	14	11, 14, 15, 17, 21, 35, 36, 37, 42, 43, 44, 45, 46, 47	Short toes and fingers, seizure, short of stature, short digits, several hypopigmented to depigmented macules on the face, multiple slightly hyperpigmented plaques on the abdomen, right arm, left thigh and leg, and right calf, multiple small hard paules on the extremities, osteoporosis with cupping deformity at articular surfaces, thyroid stimulating hormone and parathyroid hormone levels very high, calcifications in the bilateral caudate nucleus, putamen, glubus pallidus, thalamus, basal ganglia, cerebral hemipheres and cortical white matter, severe mental retardation	Bilateral cataracts and oligodontia
Agarwal et al., 2014 [26]/India	NR	CR	Polycistic ovarian syndrome (#184700)	F	21	NR	NR	Permanent	22	12, 13, 14, 15, 16, 17, 22, 23, 25, 27, 31, 32, 33, 34, 35, 37, 41, 42, 44, 45, 46, 47	NR	Slight reduction in lower anterior facial height, straight profile, mildly protuberant lips, normal nasolabial angle, deep mentolabial sulcus and hyperactivity of the mentalis muscle, oligodontia
Aminabadi et al., 2010 [27]/Iran	NR	CR	Ellis–van Creveld syndrome (#225500)	F	5	NR	NR	Primar /Permanent	6/9	52, 53, 63, 71, 73, 83/12, 31, 32, 33, 35, 41, 42, 43, 45	Short stature, extremities were plump, shortness of the limbs, hands and feet were wide and markedly deformed with sausage-shaped fingers and dysplastic fingernails, bimanual hexadactyly on the ulnar side	Hair was fine and straight, fusion of the middle portion of the upper lip to the maxillary gingival mucosal margin with absence of the mucobuccal fold, multiple accessory labiogingival frenula, conical anterior teeth, oligodontia, multiple small alveolar notches on the crest of a thin alveolar ridge, serrated appearance of the gingiva, supernumerary tooth
Ann Drum et al., 1985 [28]//United States of America	NR	CR	Rieger syndrome (#602482)	F	NR	NR	NR	Permanent	17	11, 12, 13, 14, 16, 21, 22, 23, 26, 31, 32, 33, 36, 41, 42, 45, 46	Bilateral microcornea with nasally displaced oval-shaped pupils, peripheral iris adhesions to the cornea	Oligodontia, microdontia, hypoplastic enamel, hyperplastic tissue was present where the maxillary labial frenum
Ardila and Álvarez-Martínez, 2022 [29]/Colombia	Colombia	CR	Axenfeld–Rieger syndrome (#180500)	F	5	AD	NR	Permanent	22	16, 15, 14, 13, 12, 21, 22, 25, 26, 31, 32, 33, 34, 35, 36, 37, 41, 42, 43, 44, 45, 46	Pupillary dyscoria, corectopia, blepharitis, abnormalities in the iris, and posterior embryotoxon. In profile analysis, there is a greater predominance of the upper third of the face with respect to the middle and lower thirds. A slightly flattened infraorbital area, prominent cheekbones, straight nasolabial angle, superior rretrochelia, resting labial seal, inferior normochelia, and a slightly flattened mentolabial angle are observed.	Hyperplastic upper labial frenulum, enamel hypoplasia, hypodontia, oligodontia, microdontia, taurodontism, conical teeth, short roots, and delayed eruption
Arora et al., 2016 [30]/India	NR	CR	Witkop’s syndrome (#189500)	M	23	NR	NR	Permanent	22	11, 12, 14, 15, 16, 17, 21, 23, 24, 25, 26, 27, 31, 32, 33, 34, 37, 41, 42, 43, 44, 47	Marked longitudinal ridging of the nail plates of the fingers, onychorrhexis, toenails affected with koilonychia, thin nail plates	Oligodontia, root stumps, deciduous dentition was hypoplastic, conical teeth
Awadh et al., 2016 [31]/Finland	Finland	CR	Blepharocheilodontic syndrome (#119580)	1: M 2: M 3: F 4: M	7.8–8.2	AD	NR	Primary/Permanent	1. 13/17 2. 6/7 3. 8/14 4. 7/17	1. 51, 52, 53, 54, 55, 61, 62, 63, 64, 65, 73, 74, 84/11, 12, 13, 14, 21, 22, 23, 24, 25, 27, 33, 34, 35, 41, 43, 44, 45 2. 51, 52, 73, 74, 75, 84/11, 12, 13, 21, 22, 34, 44 3. 51, 52, 54, 61, 62, 64, 74, 84/11, 12, 13, 14, 15, 21, 22, 23, 24, 25, 33, 34, 43, 44 4. 54, 62, 63, 64, 71, 81, 84/11, 12, 14, 15, 21, 22, 23, 24, 25, 31, 32, 33, 35, 41, 42, 43, 44	NR	Eyelash abnormalities, ectropion of the lower eyelids, bilateral cleft lip and palate, severe skeletal III malocclusion, oligodontia of primary and permanent teeth
Barber et al., 2012 [32]/United Kingdom	NR	CR	Carvajal syndrome (#605676)	F	15	NR	NR	Permanent	9	15, 17, 24, 25, 27, 35, 37, 45, 47	Hyperkeratotic skin on palms and soles, patchy dry, scaly skin, linear palmar and diffuse keratoderma with episodic plantar fissures, small white fingernails and thickened toenails.	Sparse eyebrows and eyelashes, premature root resorption with loss of primary teeth, microdontia incisors, recurrent angular cheilitis, granulomatous, furrowed, folded and cobblestone appearance of bilateral mucosa and fissured dorsum of tongue, episodes of generalized oral soreness twice a year, oligodontia
Bekiesinska-Figatowska et al., 2009 [33]/Poland	NR	CR	4H syndrome (#612440)	F	25	NR	NR	Permanent	8	12, 15, 22, 24, 34, 35, 44, 45	Intellectual development was mildly delayed, hypogonadotropid hypogonadism, low level of LH and FSH	Cerebellum was atrophic, corpus callosum was thin, subarachnoid cyst in the middle cranial fossa, and oligodontia
Bergendal, 2001 [34]/Sweden	NR	CR	Hypohidrotic ectodermal dysplasia (#305100)	F	6	NR	NR	Permanent	15	13, 14, 15, 24, 25, 31, 32, 33, 34, 35, 41, 42, 43, 44, 45	Capacity to sweat is reduced	Fine sparse hair, dry and thin skin, frontal bossing, cherub-like lips, no body hair on arms and legs
Bergendal et al., 2015 [35]/Sweden	NR	CR	Hypohidrotic ectodermal dysplasia (#305100)	M	32	X-linked	NR	Permanent	24	11, 12, 14, 15, 17, 21, 22, 24, 25, 27, 31, 32, 33, 34, 35, 36, 37, 41, 42, 43, 44, 45, 46, 47	Decreased sweating capacity, hoarse voice	Light sparse hair, marked oral dryness, conical teeth, mandibular anodontia, and maxillary oligodontia
Bildik et al., 2012 [36]/Turkey	NR	CR	Hypohidrotic ectodermal dysplasia (#305100)	M	14	NR	NR	Permanent	26	12, 13, 14, 15, 16, 17, 22, 23, 24, 25, 26, 27, 31, 32, 33, 34, 35, 36, 37, 41, 42, 43, 44, 45, 46, 47	Pervasive dryness on skin, excoriated papules, attention deficits, anxiety symptoms	Slimming on hairs, maxillary hypoplasia and sunken cheeks
Blankenstein et al., 2001 [37]/United Kingdom	NR	CR	Carpenter syndrome (#201000)	M	14	AR	NR	Permanent	18	12, 14, 15, 17, 22, 24, 25, 27, 31, 32, 34, 35, 37, 41, 42, 44, 45, 47	Clinodactyly and syndactyly of the fingers, preaxial polysyndactyly of the feet, hypospadias and undescended testicles, talipes equinovarus, ventriculoseptal defect, asthma	Acrocephaly, low-set ears with malformed pinnae, flattened nasal bridge, epicanthal folds, dystopia canthorum, short neck, class III malloclusion with no crowding, and oligodontia
Cagetti et al., 2019 [38]/Italy	NR	CR	Progeroid syndrome (#612289)	F	8	NR	NR	Permanent	22	11, 12, 14, 15, 16, 17, 21, 22, 23, 24, 25, 26, 27, 33, 34, 36, 37, 43, 44, 45, 46, 47	Brachycephaly, dural malformations and vascular developmental variations, hemangioma of the cephalic segment, scoliosis, skin xerosis, onychodystrophy with hypoplasia of the distal phalanx of the finger, hypertrichosis, skin laxity and wrinkles of the neck	Prominent eyebrows, misshapen teeth with radicular anomalies, condyles appeared dysmorphic and flattened, class III occlusion, and oligodontia
Callanan et al., 2006 [39]/United Kingdom	NR	CR	Sotos syndrome (#117550)	M	10	NR	NR	Permanent	11	12, 14, 15, 22, 24, 25, 34, 35, 37, 44, 45	Atrioventricular septal defect, asthma, learning disability, and behavioural problems	Macrocrania, frontal bossing, thin receding hairline, down-slanting palpebral fissures, large ears, a flat nasal bridge, pointed chin, epicanthal folds, class I malocclusion, and oligodontia
Carvalho et al., 2013 [40]/Brazil	NR	CR	Hypohidrotic ectodermal dysplasia (#305100)	1: M 2:M	1:29 2:14	NR	NR	Permanent	1.22 2. 13	1.13, 14, 15, 16, 17, 23, 24, 25, 26, 27, 31, 32, 34, 35, 36, 37, 41, 42, 44, 45, 46, 47 2.12, 15, 16, 22, 23, 31, 32, 34, 35, 41, 42, 44, 45	Thin and dry skin, and palmoplantar hyperkeratosis	1: Sparse hair, frontal bossing, hyperpigmentation in the periorbital region, low implantation of the ear, saddle nose, and oligodontia; 2: Low implantation of the ear, scanty eyebrows, and oligodontia.
Chalabreysse et al., 2011 [41]/France	NR	CR	Carvajal/Naxos syndrome (#605676/#601214)	M	21	AD	*DSP*	Permanent	11	12, 15, 17, 22, 25, 27, 35, 37, 44, 45, 47	Palmoplantar keratoderma, dilated cardiomyopathy, membranous ventricular septal defect	Wooly hair and oligodontia
Cho et al., 2004 [42]/China	China	CR	Incontinentia pigmenti/Block–Sulzberger syndrome (#308300)	M	8	X-linked dominant	NR	Permanent	6	15, 21, 25, 31, 44, 45	Typical inflammatory vesicles on the extremities shortly, linear verrucous, hyperkeratocic lesions on the extremities, hyperactivity, pigmentation on his trunk and limbs	Hypodontia deciduous, notch-shaped maxillary incisor, and oligodontia.
Clauss et al., 2014 [43]/France	NR	CR	Hypohidrotic ectodermal dysplasia (#305100)	M	NR	NR	*WNT10A*	Permanent	15	13, 14, 15, 16, 17, 23, 24, 25, 26, 27, 34, 35, 43, 44, 45	NR	Dry eyes, otitis, microdontia, cone-shaped teeth, and oligodontia
Cogulu and Ertugrul, 2008 [44]/Turkey	NR	CR	Oculofaciocardiodental syndrome (#300166)	F	12	X-linked dominant	NR	Permanent	10	12, 15, 22, 25, 31, 32, 35, 41, 42, 45	Mild cardiomegaly	Bilateral congenital cataracts, microphtalmia in the right eye, left exotropia at birth, bulbous and bifid nose, delayed eruption of the primary and permanent dentition, retained primary teeth, radiculomegaly in permanent maxillary incisorsa, and oligodontia
Cogulu et al., 2008 [45]/Turkey	NR	CR	Kabuki syndrome (#147920)	F	11	NR	NR	Permanent	18	13, 14, 15, 17, 22, 23, 24, 25, 27, 32, 33, 35, 37, 42, 43, 44, 45, 47	Mental retardation, skeletal anomalies, atrial septal defect.	Highly arched eyebrows, long palpebral fissures, eversion of the lower eyelids, large ears, flat nasal tip, cleft palate, abnormal tooth shape, widely spaced teeth, taurodont molar, and oligodontia
Costa et al., 2016 [46]/Brazil	Brazilian	CR	Zika virus syndrome (#448237)	M	3-5	NR	NR	Primary/Permanent	12 20	1.51, 53, 55, 61, 63, 64, 65, 71,73,75, 81, 85 2.13, 14, 15, 17, 22, 24, 27, 31, 32, 33, 34, 35, 36, 37, 41, 42, 43, 44, 45, 47	Cerebral palsy, foot syndactyly, and ventriculomegaly.	Mild epicanthal folds with triangular shaped facies, mild frontal bossing, fine lips, midface hypoplasia with shortened philtrum, bilateral and low- set posteriorly rotated ears small chin, and oligodontia
Dall’oca et al., 2008 [47]/Italy	NR	CR	Hypohidrotic ectodermal dysplasia (#305100)	M	11	X-linked dominant	NR	Primary	17	51, 52, 53, 55, 61, 62, 63, 65, 71, 72, 73, 74, 75, 81, 82, 83, 85	Dry skin, delicate and prone rashes, thin, light blond and wooly hair, poor eyelashes and eyebrows, heat intolerance, hyper-pigmented in the periocular area and around the mouth	Slight depression of the bridge of the nose, malformed upper molars, conoid cusps and oligodontia
Devadas et al., 2005 [48]/India	India	CR	Witkop’s syndrome (#189500)	F	5	NR	NR	Permanent	10	12, 22, 23, 25, 31, 32, 33, 41, 42, 43	Short stature, hypoplastic fingernails, thin and easily chipped toenails	Scanty eyebrows, slightly drooping eyelids, prominent epicanthal folds, microdontia, and oligodontia
Downing and Welbury, 1992 [49]/United Kingdom	NR	CR	Trichorhinophalangeal syndrome (#190350)	M	8	NR	NR	Permanent	8	12, 16, 22, 26, 31, 32, 41, 42	Soft nails, dry skin, broad terminal phalanges of fingers and toes, short stature, cone-shaped epiphyses	Sparse blond hair, prominent ears, broad pear-shaped nose, a long philtrum, thin upper lip, class III malocclusion, and oligodontia
Dunbar et al., 2015 [50]/United Kingdom	NR	CR	Axenfeld–Rieger syndrome (#180500)	M	10	NR	NR	Permanent	8	11, 12, 13, 21, 22, 32, 35, 45	NR	Class III malocclusion and oligodontia
Emral and Akcam, 2009 [51]/Turkey	NR	CR	Noonan syndrome (#163950)	M	13	AD	NR	Permanent	7	12, 22, 27, 34, 35, 45, 46	Short stature, asymmetry between the left and right thorax, pectus excavatum, widely spaced nipples	Facial dismorphism, bilateral telecanthus, myopia, 13 diopter, low neck-hair line, mane neck, high palatal vault, drooping eyelids, asymmetric smile, low-set ears, thick helix of the ear, small, upturned nose, broad forehead, deeply grooved philtrum, and oligodontia
Fan et al., 2019 [52]/China	NR	CR	Axenfeld–Rieger syndrome (#180500)	1:F 2:F 3:F 4:F	7-29	AD	*PITX2*	Primary/Permanent	1. 10 2. 9 3. 22 4. 9/19	1. 11, 12, 13, 14, 21, 22, 23, 27, 33, 43 2.11, 13, 21, 23, 31, 41, 42, 43, 45 3.11, 12, 13, 14, 15, 17, 21, 22, 23, 24, 25, 27, 31, 32, 33, 34, 35, 41, 42, 43, 44, 45 4.51, 52, 54, 55, 61, 62, 63, 64, 65/11, 12, 13, 14, 15, 16, 17, 21, 22, 23, 24, 25, 27, 31, 33, 35, 41, 45, 47	Umbilical stump abnormality	Severe ocular anterior chamber anomalies, corneal opacity, iridocorneal adhesion, angle cosure, iris hypoplasia, corectopia, glaucoma, complete blindness, and oligodontia
Ghosh et al., 2019 [53]/India	India	CR	Microcephalic osteofysplastic dwarfism type II (#210720)	M	8	AR	*PCNT*	Permanent	14	12, 13, 14, 15, 17, 22, 24, 31, 33, 34, 35, 43, 44, 45	Hyperpigmented skin, hypotrichosis of scalp, eyebrow, eyelashes, thin upper and lower extremities with clinodactyly, bilateral flat foot, high-pitched voice, mild thracolumbar scoliosis	Micrognathia, large prominent eyes, beak-shaped nose with deviated nasal septum, conical roots, horizontal growth pattern with skeletal deep bite, craniostenosis, and oligodontia.
Gupta, 2012 [54]/India	India	CR	Oromandibular limb hypogenesis syndrome type I B (#103300)	M	28	NR	NR	Permanent	10	12, 15, 22, 25, 31, 32, 35, 41, 42, 45	Oropharyngeal isthmus absence of the palatoglossal arches	Medium height with a long narrow face, fused eyebrows, low-set ears, broad nasal alae, saddle nose, pointy tapering chin, thin lips, complete absence of tongue, and oligodontia
Hasan et al., 2019 [55}/India	NR	CR	Hypohidrotic ectodermal dysplasia (#305100)	F	19	AR	NR	Permanent	15	11, 12, 21, 22, 23, 31, 32, 35, 36, 41, 42, 43, 44, 45, 46	Heat intolerance with raised body temperature and reduced perspiration, short stature, normal-shaped fingers, and broad, short, and wide-spaced toes	Reduced anterior facial height and mild hyperpigmented, wrinkled periorbital skin, receding hairline, thin, lusterless hair with premature graying, prognathic mandible, and oligodontia
Hassona et al., 2018 [56]/Jordan	NR	CR	Sanjad–Sakati syndrome (#241410)	F	15	NR	*TBCE*	Permanent	14	12, 13, 14, 15, 22, 23, 24, 25, 33, 34, 35, 42, 44, 45	Hypocalcemia, hyperphosphatemia, low parathyroid hormone level, small hands and feet with short and thin digits, microcephaly	Low set ears, beaked nose, retrognathic mandible, thin lips, incomplete root formation, taurodontism, and oligodontia
Hattab et al., 1996 [57]/Iran	Jordan	CR	Grebe chondrodysplasia (#200700)	M	9	NR	NR	Permanent	25	11, 12, 13, 14, 15, 17, 21, 22, 23, 24, 25, 27, 31, 32, 33, 34, 35, 36, 37, 41, 42, 43, 44, 45, 47	Short stature, deformed limbs, difficulty walking, short and deformed arms, disproportionately short fingers, fingertips were budlike, small, and with dysplastic nails, markedly short and asymmetric legs, talipes equinovarus, irregular and bulbous toes, left ulna and fibula were hypoplastic, right fibula absent	Asymmetric and dysmorphic with a prominent forehead, flat occiput, depressed middle third of the face, ocular hypertelorism with antimongoloid slanting, severe loss of hearing, depressed bridge of the nose, frontal bossing, angular highly set orbital plates, hypoplastic maxilla, prognathic mandible, and oligodontia.
Hattab et al., 1997 [58]/Sweden	NR	CR	Polycystic ovarian syndrome (#184700)	1: F 2: F	1:21 2:18	NR	NR	Permanent	1.26 2.22	1. 12, 13, 14, 15, 16, 17, 22, 23, 24, 25, 26, 27, 31, 32, 33, 34, 35, 36, 37, 41, 42, 43, 44, 45, 46, 47 2.12, 13, 15, 16, 17, 22, 23, 24, 25, 26, 27, 31, 32, 33, 34, 35, 37, 41, 42, 44, 45, 46	Enlarged ovaries with multiple cystic-like lesions	Long and coarse hair and oligodontia
Hingston et al., 2006 [59]/United Kingdom	NR	CR	Hurler’s syndrome (#607014)	F	11	NR	NR	Permanent	8	14, 15, 24, 25, 34, 35, 44, 45	Short stature	Large head, short neck, corneal clouding, depressed nasal bridge, broad nasal tip, long upper lip with relative flattening of the philtrum, and oligodontia
Jain et al., 2010 [60]/India	NR	CR	Hypohidrotic ectordemal dysplasia (#305100)	M	13	NR	NR	Permanent	21	12, 13, 14, 15, 17, 22, 23, 24, 25, 27, 31, 32, 34, 35, 36, 37, 41, 42, 43, 44, 45	Maculopapular eruptions, onchodysplasia	Protuberant supraorbital ridge, frontal bossing, sparse hairs, scant eyelashes and eyebrows, saddle nose, conical-shaped teeth, hypoplastic teeth, and oligodontia
Jain et al., 2012 [61]/India	NR	CR	Hypohidrotic ectodermal dysplasia (#305100)	M	11	NR	NR	Permanent	25	13, 14, 15, 16, 17, 21, 22, 24, 25, 26, 27, 31, 32, 33, 34, 35, 36, 37, 41, 42, 43, 44, 45, 46, 47	Lack of sweating, dryness of skin, raised body temperature	Sparse hair, frontal bossing, prominent supra orbital ridges, depressed nasal bridge, sunken cheeks, protuberant lips, decreased lower facial height, absence of saliva, dry oral mucosa, and oligodontia
Kale et al., 2013 [62]/India	NR	CR	Achondroplasia (#100800)	F	16	NR	NR	Permanent	18	12, 14, 16, 22, 24, 26, 31, 32, 33, 34, 35, 37, 41, 42, 43, 44, 45, 47	Short stature, rhizomelic shortening of the arms and legs, lumbar lordosis, prominent buttocks, stubby fingers with trident hand, brachycephaly	Shortening of the skull base with frontal bossing, flat nasal bridge, retruded maxilla, hypertonic lips, and oligodontia
Kantaputra et al., 2014 [63]/Thailand	Thailand	CR	Tricho-odonto-onychodermal dysplasia (#275980)	1: F 2: F 3: F	16-27	AR	*WNT10A*	Permanent	1.26 2.21 3.12	1. 11, 12, 13, 14, 16, 17, 21, 22, 23, 24, 26, 27, 31, 32, 33, 34, 35, 36, 37, 41, 42, 43, 44, 45, 46, 47 2.12, 13, 14, 15, 17, 22, 23, 24, 25, 31, 32, 33, 34, 37, 41, 42, 43, 44, 45, 46, 47 3. 12, 14, 16, 22, 27, 31, 32, 35, 41, 42, 45, 46	Dystrophic fingernails and toenails, palmoplantar keratoderma, dry skin, skin improved with age	Straight hair during childhood, slow growing scalp hair, dysplastic hair follicle, trichorrhexis nodosa-like hair anomaly, facial freckles, barrel-shaped mandibular incisors, and oligodontia
Kaul and Reddy, 2008 [64]/India	NR	CR	Hypohidrotic ectodermal dysplasia (#305100)	M	14	NR	NR	Permanent	24	12, 13, 14, 15, 16, 17, 22, 23, 24, 25, 26, 27, 31, 32, 33, 34, 35, 37, 41, 42, 43, 44, 45, 47	Dry skin, frequent bouts of fever, intolerant to heat, scanty body hair	Frontal bossing, scanty scalp hair, periorbital pigmentation, low-set ears, depressed nasal, protuberant lips, dry mucosa, malformed incisors impacted, and oligodontia
Kawamoto, Motohashi and Ohyama, 2004 [65]/Japan	Japan	CR	Oculofaciocardiodental syndrome (#300166)	F	13	X-linked dominant	NR	Permanent	7	12, 15, 24, 31, 32, 35, 41	Cardiac anomalies	Congenital cataract, dental root gigantism, and oligodontia
Khabour et al., 2010 [66]/Jordan	Jordan	CR	Hypohidrotic ectodermal dysplasia (#305100)	M	11	X-linked	*EDA*	Permanent	17	12, 14, 15, 17, 22, 24, 25, 27, 32, 34, 35, 36, 37, 42, 44, 45, 47	Damaged eccrine glands (anhidrosis)	Sparse scalp hair, absent eyebrows and eyelashes, periorbital wrinkling, saddle nose, protuberant lips, abnormally shaped upper and lower permanent teeth, and oligodontia.
Khurana et al., 2012 [67]/Jordan	NR	CR	Witkop syndrome (#189500)	M	20	AD	*MSX1*	Permanent	11	11, 12, 17, 21, 22, 27, 31, 35, 37, 44, 47	Abnormalities of both toenails and fingernails	Sparse scalp hair, smaller mandibles, and oligodontia
Kinyó et al., 2014 [68]/Hungary	Hungary	CR	Christ–Siemens–Touraine syndrome (#305100)	M	35	X-linked	*EDA*	Permanent	25	11, 14, 15, 16, 17, 21, 22, 24, 25, 26, 27, 31, 32, 33, 34, 35, 36, 37, 41, 42, 43, 44, 45, 46, 47	Dryness of the skin, eyes, airways, and mucous membranes	Missing eyebrows and eyelashes, sparse hair, and oligodontia
Kishore et al., 2014 [69]/India	India	CR	Hypohidrotic ectodermal dysplasia (#305100)	1: M 2: M	1:20 2:25	X-linked recessive	NR	Permanent	24	17, 16, 15, 14, 13, 23, 24, 25, 26, 27, 37, 36, 35, 34, 33, 32, 31, 41, 42, 43, 44, 45, 46, 47	Skin was dry and wrinkled with no nail dystrophy	Frontal bossing, scarce eyebrows and eyelashes, thinning of scalp hair, prominent supraorbital ridges, depressed nasal bridge, thick and prominent lips with a concave profile, and oligodontia
Kobayashi et al., 2022 [70]/Japan	NR	CR	Hypohidrotic ectodermal dysplasia (#305100)	F	24	AD	*EDA*	Permanent	25	11, 12, 13, 14, 15, 17, 21, 22, 23, 24, 25, 27, 31, 32, 33, 34, 35, 37, 41, 42, 43, 44, 45, 46, 47	Mammary glands hypoplastic and the surfaces of her palms and soles glossy and partially eroded	The lower face was short, the oral fissure was narrow, the middle face sank, and the lips protruded, resembling the so-called prematurely aged face in the profile, scalp hair thin and soft, and oligodontia
Kozma, et al., 1999 [71]/United States of America	Saudia Arabia	CR	Wolf–Hirschhorn syndrome (#194190)	M	11	NR	NR	Permanent	9	14, 24, 31, 34, 35, 37, 41, 44, 45	NR	Oligodontia
Kramer et al., 2005 [72]/Germany	NR	CR	Hypohidrotic ectodermal dysplasia (#305100)	M	3	NR	NR	Primary /Permanent	1. 18/26	52, 53, 54, 55, 62, 63, 64, 65, 71, 72, 73, 74, 75, 81, 82, 83, 84, 85 / 12, 13, 14, 15, 16, 17, 22, 23, 24, 25, 26, 27, 31, 32, 33, 34, 35, 36, 37, 41, 42, 43, 44, 45, 46, 47	Dry hypopigmented scaly skin	Hair was thin, sparse, and blond with sparse eyelashes and eyebrows, as well as oligodontia
Kroigard et al., 2016 [73]/Denmark	NR	CR	Odonto-onychodermal dysplasia (#257980)	F	75	NR	*WNT10A*	Permanent	26	12, 13, 14, 15, 16, 17, 22, 23, 24, 25, 26, 27, 31, 32, 33, 34, 35, 36, 37, 41, 42, 43, 44, 45, 46, 47	Dry skin, slightly dystrophic and brittle finger and toe nails with spooning.	Erythematous plaques on both cheeks and the nose, facial basal cell carcinomas, and oligodontia
Levy et al., 2020 [74]/France	NR	CR	Hypohidrotic ectodermal dysplasia (#305100)	1: F 2:M	1:23 2:6	X-linked	*LEF1*	Primary/Permanent	1. 7/19 2. 19	1. 55, 53, 63, 65, 73, 74, 75/17, 16, 15, 14, 13, 24, 25, 26, 27, 35, 34, 33, 32, 31, 41, 42, 43, 44, 45 2. 16, 15, 14, 12, 22, 24, 27, 37, 35, 34, 33, 32, 31, 41, 42, 43, 44, 45, 47	NR	1: Sparse, fine hair, sparse eyelashes and eyebrows, and oligodontia; 2: Sparse, fine hair, sparse eyelashes and eyebrows, microdontia, and oligodontia.
Liedén et al., 2014 [75]/Sweden	NR	CR	AT-rich sequence-binding protein 2 gene (#608148)	M	20	NR	NR	Permanent	10	11, 12, 13, 14, 24, 25, 33, 35, 43, 44	Impairment of fine and gross motor skills, left-sided mild hemiparesis, and spasticity with increased reflexes in the upper and lower extremities bilaterally, mild–moderate osteoporosis	Tall forehead, bushy eyebrows, a prominent nose, cleft palate was noted early, with surgical correction at the age of 11 months, conical front teeth, generally large teeth crowns and with extremely short, malformed roots and oligodontia
Lin et al., 2022 [76]/China	NR	CR	Blepharocheilodontic syndrome (#119580)	1:M 2:F	9 35	AD	*CDH1*	Permanent	1.14 2.19	1. 11, 12, 13, 14, 15, 21, 22, 23, 24, 25, 31, 34, 41, 44 2. 11, 12, 13, 15, 21, 22, 23, 24, 25, 31, 32, 33, 34, 35, 41, 42, 43, 44, 45	Euryblepharon, ectropion, distichiasis	High frontal hairline, broad forehead, and oligodontia
Martinho, et al., 2019 [77]/Portugal	NR	CR	Oculofaciocardiodental syndrome (#300166)	F	26	X-linked dominant	NR	Permanent	10	13, 15, 16, 22, 24, 26, 27, 36, 37, 46	Mild cardiomegaly, a misalignment of her thumbs, valgus foot, and prolapsed mitral valve	Long, narrow face (dolichocephalous) with a bifid tip of the nose and very deep-set eyes with cataract, and oligodontia
Marvin et al., 2011 [78]/United States of America	NR	CR	Hypohidrotic ectodermal dysplasia (#305100)	F	35	NR	*AXIN2*	Permanent	11	13, 17, 23, 27, 31, 32, 37, 41, 42, 43, 47	NR	Hypognathia, fine scalp hair, very sparse eyebrows, slightly up-slanting palpebral fissures, malar hypoplasia, broad nasal bridge, fine scalp hair, slightly up-slanting palpebral fissure, and oligodontia
Murata et al., 2019 [79]/Japan	Japan	CR	Basal cell nevus syndrome (#109400)	F	6	NR	*PTCH1*	Permanent	10	12, 14, 15, 22, 24, 25, 31, 34, 35, 45	Cheiloplasty, palatoplasty, and surgical excision of cardiac fibroma and palmar pits	Ocular hypertelorism, bilateral cleft lip and palate, and oligodontia
O’Dwyer and Jones, 2005 [80]/United Kingdom	NR	CR	Axenfeld–Rieger syndrome (#180500)	F	10	NR	NR	Permanent	11	11, 12, 13, 21, 22, 23, 27, 32, 35, 42, 45	NR	Severe hypodontia, microdontia, short root, and oligodontia
Pipa Vallejo et al., 2008 [81]/Turkey	NR	CR	Hypohidrotic ectodermal dysplasia (#305100)	M	5	X-linked recessive	NR	Primary	15	54, 53, 51, 63, 64, 65, 75, 74, 73, 72, 71, 81, 83, 84, 85	Fine dry skin on the palms and soles	Parse, fine silky hair, thinly covering the scalp, thin eyebrows, folded lower eyelid, narrow nose at the tip, thick lower lip, and oligodontia
Reiche et al., 2014 [82]/United States of America	NR	CR	Down’s syndrome (#190685)	NR	18	NR	NR	Permanent	6	12, 17, 22, 27, 31, 41	NR	Macroglossia and oligodontia
Retna and Sockalingam, 2003 [83]/Malaysia	China	CR	Blepharocheilodontic syndrome (#119580)	F	5	NR	NR	Permanent	20	11, 13, 14, 15, 17, 21, 22, 24, 25, 31, 32, 33, 34, 35, 37, 41, 42, 43, 44, 45	Bilateral eye anomalies that include ectropion of the lower eyelids, lagophthalmos, hypertelorism, distichiasis and euryblepharon, and scalp hair was sparse and straight	Bilateral cleft lip and palate, microstomia, and oligodontia and microdontia with conical-shaped teeth
Richieri-Costa et al.,1993 [84]/Canada	NR	CR	Carpenter syndrome (#201000)	F	6	NR	NR	Permanent	26	12, 13, 14, 15, 16, 17, 22, 23, 24, 25, 26, 27, 31, 32, 33, 34, 35, 36, 37, 41, 42, 43, 44, 45, 46, 47	Hypertelorism, small hands, brachydactyly, camptodactyly of the left third finger, small feet, proximal cutaneous syndactyly of toes, and clinodactyly of toes	Thick neck, dolichoplagiomacrocephaly, prominent forehead, blow nasal bridge, hypoplastic alveolar ridges, and oligodontia
Rizos et al., 1998 [85]/United States of America	NR	CR	Mobius syndrome (#157900)	F	17	NR	NR	Permanent	8	11, 17, 23, 24, 25, 26, 27, 32	NR	Facial hemiplegia and oligodontia
Rock and McLellan, 1990 [86]/United Kingdom	NR	CR	Klinefelter syndrome (NR)	M	8	NR	NR	Permanent	11	12, 14, 15, 22, 24, 25, 34, 35, 37, 44, 45	NR	Broad nasal bridge and hypertelorism
Shen et al., 2011 [87]/China	China	CR	Ellis–van Creveld syndrome (#225500)	F	6	AR	*EVC2*	Permanent	8	12, 13, 22, 23, 31, 32, 41, 42	Postaxial polydactyly of both hands, syndactyly of the toes and nail dysplasia, and abnormalities of the knee joints	Hipodontia and oligodontia
Siddiqui et al., 2021 [88]/India	NR	CR	Axenfeld–Rieger syndrome(#180500)	F	11	AD	*PITX2*	Permanent	14	11, 12, 14, 15, 16, 17, 21, 22, 25, 31, 32, 35, 41, 42	Ophthalmic records revealed anterior segment dysgenesis with corectopia (displacement of the pupil) and polycoria (multiple pupils)	Maxillary hypoplasia with poor upper lip support and thick everted lower lip and oligodontia
Sikora et al., 2016 [89]/Australia	NR	CR	Juvenile nephronophtisis (#256100)	F	11	NR	*NPHP1*	Permanent	12	12, 17, 23, 26, 27, 31, 32, 33, 37, 41, 42, 47	Short hands and feet and genu valgum	Dysmorphic face—broad nose, large mouth, small chin, bilateral fusion of the 1 and 2 upper teeth, and oligodontia
Singh et al., 2001 [90]/India	India	CR	Floating-Harbor syndrome (#136140)	M	14	NR	*SRCAP*	Permanent	8	12, 22, 23, 31, 32, 33, 41, 43	Bilateral inguinal testis, cryptorchidism, hypospadias, and congenital absence of right kidney	Leptoproscopic facial form, deep-set eyes, long eyelashes, ptosis of upper eyelids, reduced malar prominence, low-set ears with outwardly rotated ear lobes, broad and bulbous nose, low-hanging columella, smooth philtrum, reduced dry vermilion of the upper lip, slightly everted lower lip, ankyloglossia with thick frenum and restricted tongue movements, and oligodontia
Subramaniam and Neeraja, 2008 [91]/India	NR	CR	Witkop’s syndrome (#189500)	M	14	NR	NR	Permanent	24	12, 13, 14, 15, 17, 22, 23, 24, 25, 27, 31, 32, 33, 34, 35, 36, 37, 41, 42, 43, 44, 45, 46, 47	NR	Oligodontia
Suda et al., 2010 [92]/Japan	NR	CR	Hypohidrotic Ectodermal dysplasia (#305100)	M	5	NR	*EDARADD*	Permanent	27	11, 12, 13, 14, 15, 16, 17, 21, 22, 23, 24, 25, 26, 27, 31, 32, 33, 34, 35, 36, 37, 41, 42, 43, 44, 45, 47	Unable to sweat	Hair and eyebrows were sparse, oligondontia
Sultan et al., 2020 [93]/India	NR	CR	Down syndrome (#190685)	M	12	NR	NR	Permanent	8	12, 22, 31, 35, 37, 41, 42, 45	Short stature and mentally challenged	Saddle nose deformity, hypertelorism, midface hypoplasia with retruded maxilla and protruded mandible, including the intra-oral aspects of the high arch palate, macroglossia of the tongue, and oligodontia
Sun et al., 2019 [94]/China	China	CR	Incontinentia pigmenti (#308300)	F	15	X-linked dominant	*IKBKG*	Permanent	11	15, 17, 22, 24, 25, 34, 35, 37, 44, 45, 46	NR	Oligodontia
Talasila et al., 2017 [95]/India	India	CR	Acromelia-oligodontia syndrome (NR)	M	18	NR	NR	Permanent	6	31, 33, 35, 36, 37, 41	The forelimbs show rudimentary thumbs and acromelia	High forehead, zygomatic and mandibular hypoplasia on the left, deviation of the chin to the left, blepharophimosis, ankyloglossia, and oligodontia
Tanboga et al., 2001 [96]/Turkey	India	CR	Incontinentia pigmenti (#308300)	F	6	X-linked dominant	NR	Primary/Permanent	8/17	52, 62, 64, 72, 75, 81, 82, 85/12, 13, 14, 15, 17, 22, 23, 24, 25, 27, 32, 35, 37, 41, 42, 45, 47	NR	Oligodontia
Tanboga, et al., 1992 [97]/Turkey	NR	CR	Hypohidrotic ectodermal dysplasia (#305100)	M	7	NR	NR	Permanent	8	11, 16, 21, 26, 32, 36, 42, 46	Hands and feet characterized by syndactyly of the digits and absent, hypoplastic, or fused phalanges and metacarpals	Hair and eyebrows were sparse, oligondontia
Tao et al., 2010 [98]/United States of America	NR	CR	Beare–Stevenson syndrome (#123790)	M	6	AD	*FGFR2*	Permanent	9	15, 25, 31, 32, 35, 41, 42, 44, 45	Cutis gyrate, acanthosis nigricans, skin tags, ear defects, anogenital anomalies, and a prominent umbilical stump	Midface hypoplasia, natal teeth, and oligodontia
Tosun et al., 2006 [99]/Turkey	NR	CR	Apert syndrome (#101200)	M	4	AD	NR	Primary/ Permanent	8/8	52, 55, 62, 65, 72, 75, 82, 85/14, 15, 24, 25, 34, 35, 44, 45	Syndactyly of hands and feet digit	Displayed midface hypoplasia, a flat/steep forehead, an extremely short, depressed nose, proptosis, trapezoidal shape of mouth, tongue appeared excessively large, pseudo cleft palate, and oligodontia
Tuna et al., 2009 [100]/Istanbul	NR	CR	Hallermann–Streiff (HSS) syndrome (#234100)	F	10	NR	NR	Permanent	16	12, 14, 15, 22, 24, 25, 31, 32, 33, 34, 35, 41, 42, 43, 44, 45	Decreased oropharyngeal airway	Micrognathia, sparse hair, eyebrows, eyelashes, cutaneous atrophy, congenital bilateral cataracts, ptosis, horizontal nystagmus, blue sclera, mildly down-slanting palpebral fissures, lateral collapse of the middle and lower lateral cartilages at the nose, hypertrophy of the turbinates, excessive long soft palate, nasal septum deviated, open bite, moderate anterior crowding and malformed teeth, bilateral posterior crossbite, prominent tongue-thrust swallowing, excessive vertical dimension of the lower third of the face, and oligodontia
Tuna et al., 2012 [101]/Turkey	NR	CR	Kabuki syndrome (#147920)	M	5	AD	NR	Permanent	10	12, 14, 22, 24, 32, 33, 35, 42, 43, 45	Short fifth finger, small middle phalanges, and hyperelastic finger joints	Long and everted lateral and palpebral fissures, dysplastic prominent ears, blue sclera, a short nasal septum, depressed nasal tip, hypermobility of the joints, and oligodontia
Vasudevan and Sinha, 2023 [102]/India	NR	CR	Odonto-onychiadermal dysplasia (#257980)	M	19	NR	NR	Permanent	24	11, 12, 13, 14, 15, 16, 17, 22, 23, 24, 25, 26, 31, 32, 33, 34, 35, 37, 41, 42, 43, 44, 45, 47	Hyperkeratosis on the pal mar aspect of both hands, dystrophy of the toe nails, and ichthyosis of posterior aspect of legs	Oligodontia
Waldron et al., 2010 [103]/Ireland	Ireland	CR	Axenfeld–Rieger syndrome (#180500)	F	7	AD	NR	Permanent	14	11, 12, 13, 21, 22, 23, 31, 32, 33, 35, 41, 42, 43, 45	NR	Oligodontia
Yin et al., 2013 [104]/China	NR	CR	Hypohidrotic ectodermal dysplasia (#305100)	M	5	NR	*EDA*	Permanent	26	12, 13, 14, 15, 16, 17, 22, 23, 24, 25, 26, 27, 31, 32, 33, 34, 35, 36, 37, 41, 42, 43, 44, 45, 46, 47	NR	Oligodontia
Zidane and Alloussi, 2022 [105]/Marrocos	NR	CR	Dubowitz syndrome (#223370)	M	6	AR	*NR*	Permanent	15	15, 14, 13, 12, 22, 23, 24, 25, 35, 34, 32, 31, 41, 44, 45	NR	Had a high-pitched voice, mild mental retardation, and oligodontia

CR: case report; SC: series of cases; AD: autosomal dominant; AR: autosomal recessive; X-LINKED.

## Data Availability

No new data were created or analyzed in this study. Data sharing is not applicable.

## Supplementary Materials

The following supporting information can be downloaded at https://www.mdpi.com/article/10.3390/dj11120279/s1: Supplementary Table S1. Search strategies with appropriate key words and MeSH terms. Supplementary Table S2. Risk of bias assessment of the individual articles included (n:83). Risk of bias was categorized as High when the study reaches up to 49% score “yes”, Moderate when the study reached 50% to 69% score “yes”, and Low when the study reached more than 70% score “yes”. Supplementary Table S3. Biological processes characterized by the list of altered genes in syndromes with oligodontia. Supplementary Table S4. Activated pathways characterized with syndromes with oligodontia.
