# Supplementary material for: Oligodontia in the Clinical Spectrum of Syndromes: A Systematic Review
Source: Dent J (Basel). 2023 Dec 4;11(12):279. doi: 10.3390/dj11120279 (PMC10742796; doi:10.3390/dj11120279)
Supplement: Supplementary file 1 [file dentistry-11-00279-s001.zip › dentistry-2609278-supplementary.pdf]

Supplementary Table 1. Search strategies with appropriated key words and MeSH terms.

Database	Search strategies<br>(search date: August 8, 2023)	Total
LILACS	(tw:("Syndrome oligodontia" OR "syndromes oligodontia" OR "Syndromic oligodontia" OR "oligodontia sindoromica" OR "sindrome oligodontia" OR "sindromes oligodontia" OR "sindrome oligodoncia" OR "oligodoncia sindromica" OR "sindromes oligodoncia" OR "LTBP3" OR "WNT10A" OR "EDARADD" OR "EDA" OR "IKBKG" OR "MSX1" OR "AXIN2")) AND (tw:(anodontismo OR anodontia OR Oligodontia OR "Severe hypodontia" OR anodoncia OR oligodoncia OR "hipodontia severa"))	27
Embase	('anodontia'/exp OR 'anodontia' OR 'oligodontia'/exp OR 'oligodontia' OR 'severe hypodontia') AND ('syndrome oligodontia' OR 'syndromes oligodontia' OR 'syndromic oligodontia' OR 'ltbp3' OR 'wnt10a' OR 'edaradd' OR 'eda'/exp OR 'eda' OR 'ikbkg' OR 'msx1' OR 'axin2'/exp OR 'axin2')	319
PubMed	("Syndrome oligodontia" OR "syndromes oligodontia" OR "Syndromic oligodontia" OR "LTBP3" OR "WNT10A" OR "EDARADD" OR "EDA" OR "IKBKG" OR "MSX1" OR "AXIN2") AND ("anodontia"[MeSH Terms] OR "anodontia" OR "Oligodontia" OR "Severe hypodontia")	1300
Scopus	TITLE-ABS-KEY("Syndrome oligodontia" OR "syndromes oligodontia" OR "Syndromic oligodontia" OR "LTBP3" OR "WNT10A" OR "EDARADD" OR "EDA" OR "IKBKG" OR "MSX1" OR "AXIN2") AND TITLE-ABS-KEY("anodontia" OR "Oligodontia" OR "Severe hypodontia")	412
Livivo	("Syndrome oligodontia" OR "syndromes oligodontia" OR "Syndromic oligodontia" OR "LTBP3" OR "WNT10A" OR "EDARADD" OR "EDA" OR "IKBKG" OR "MSX1" OR "AXIN2") AND ("anodontia" OR "Oligodontia" OR "Severe hypodontia")	151
Web of Science	TS=(Syndrome oligodontia OR syndromes oligodontia OR Syndromic oligodontia OR LTBP3 OR WNT10A OR EDARADD OR EDA OR IKBKG OR MSX1 OR AXIN2) AND TS=(anodontia OR Oligodontia OR Severe hypodontia)	360

Google Scholar	("Syndrome oligodontia" OR "syndromes oligodontia" OR "Syndromic oligodontia" OR "LTBP3" OR "WNT10A" OR "EDARADD" OR "EDA" OR "IKBKG" OR "MSX1" OR "AXIN2") AND ("anodontia"[MeSH Terms] OR "anodontia" OR "Oligodontia" OR "Severe hypodontia")	74
Open Grey	Oligodontia	7

Supplementary Table 2. Risk of bias assessment of the individual articles included (n:84)

Authors / Year	1. Were patient's demographic characteristics clearly described?	2.Was the patient's history clearly described and presented as a timeline?	3.Was the current clinical condition of the patient on presentation clearly described?	4.Were diagnostic tests or assessment methods and the results clearly described?	5.Was the intervention(s) or treatment procedure(s) clearly described?	6.Was the post-intervention clinical condition clearly described?	7.Were adverse events (harms) or unanticipated events identified and described?	8.Does the case report/serius provide takeaway lessons?
Abdulla et al., 2019	Unclear	Yes	Yes	Yes	Not applicable	Not applicable	Not applicable	Yes
Abs et al., 1994	Yes	Yes	Yes	no	Not applicable	Not applicable	Not applicable	Yes
Aditya et al., 2011	Yes	Yes	Yes	Yes	Not applicable	Not applicable	Not applicable	Yes
Agarwal et al., 2014	Yes	Yes	Yes	Yes	Not applicable	Not applicable	Not applicable	Yes
Aminabadi et al., 2010	Yes	Yes	Yes	Yes	Not applicable	Not applicable	Not applicable	Yes
Ann Drum et al., 1985	Yes	Yes	Yes	Yes	Not applicable	Not applicable	Not applicable	Yes
Ardila and Álvarez-Martínez, 2022	No	Yes	Yes	Yes	Not applicable	Not applicable	Not applicable	Yes
Arora et al., 2016	Yes	Yes	Yes	Yes	Not applicable	Not applicable	Not applicable	Yes
Awadh et al., 2016	Yes	Yes	Yes	Yes	Not applicable	Not applicable	Not applicable	Yes
Barber et al., 2012	Yes	Yes	Yes	Yes	Not applicable	Not applicable	Not applicable	Yes

Bekiesinska-Figatowska et al., 2010	Yes	Yes	Yes	Yes	Not applicable	Not applicable	Not applicable	Yes
Bergendal, 2001	Yes	Yes	Yes	Unclear	Not applicable	Not applicable	Not applicable	Yes
Bergendal et al., 2015	Yes	Yes	Yes	Yes	Not applicable	Not applicable	Not applicable	Yes
Bildik et al., 2012	Yes	Yes	Yes	Yes	Not applicable	Not applicable	Not applicable	Yes
Blankenstein et al., 2001	Yes	Yes	Yes	Yes	Not applicable	Not applicable	Not applicable	Yes
Cagetti et al., 2019	Yes	Yes	Yes	Unclear	Not applicable	Not applicable	Not applicable	Yes
Callanan et al., 2006	Yes	Yes	Yes	Unclear	Not applicable	Not applicable	Not applicable	Yes
Carvalho et al., 2013	Yes	no	Yes	no	Not applicable	Not applicable	Not applicable	Yes
Chalabreysse et al., 2011	Yes	Yes	Yes	Yes	Not applicable	Not applicable	Not applicable	Yes
Cho et al., 2004	Yes	Yes	Yes	Yes	Not applicable	Not applicable	Not applicable	Yes
Clauss et al., 2014	Yes	Yes	Yes	Yes	Not applicable	Not applicable	Not applicable	Yes
Cogulu and Ertugrul, 2007	Yes	no	Yes	no	Not applicable	Not applicable	Not applicable	Yes
Cogulu and Ertugrul, 2007	Yes	no	Yes	no	Not applicable	Not applicable	Not applicable	Yes
Costa et al., 2022	Yes	No	Yes	No	Not applicable	Not applicable	Not applicable	Yes
Dall’oca et al., 2008	Yes	Unclear	Yes	Yes	Not applicable	Not applicable	Not applicable	Yes

Devadas et al., 2005	Yes	Yes	Yes	Yes	Not applicable	Not applicable	Not applicable	Yes
Downing and Welbury, 1992	Yes	Yes	Yes	Unclear	Not applicable	Not applicable	Not applicable	Yes
Dunbar et al., 2015	Yes	Yes	Yes	Yes	Not applicable	Not applicable	Not applicable	Yes
Emral and Akcam, 2009	Yes	Yes	Yes	Yes	Not applicable	Not applicable	Not applicable	Yes
Fan et al., 2019	Yes	Yes	Yes	Yes	Not applicable	Not applicable	Not applicable	Yes
Ghosh et al., 2019	Yes	Yes	Yes	Yes	Not applicable	Not applicable	Not applicable	Yes
Gupta, 2012	Yes	Yes	Yes	no	Not applicable	Not applicable	Not applicable	Yes
Hasan et al., 2019	Yes	Yes	Yes	no	Not applicable	Not applicable	Not applicable	Yes
Hassona et al., 2018	Yes	Yes	Yes	Yes	Not applicable	Not applicable	Not applicable	Yes
Hattab et al., 1996	Yes	Yes	Yes	Unclear	Not applicable	Not applicable	Not applicable	Yes
Hattab et al., 1997	Yes	Yes	Yes	Unclear	Not applicable	Not applicable	Not applicable	Yes
Hingston et al., 2006	Yes	Yes	Yes	Yes	Not applicable	Not applicable	Not applicable	Yes
Jain et al., 2010	Yes	Yes	Yes	Yes	Not applicable	Not applicable	Not applicable	Yes
Jain et al., 2012	Yes	Yes	Yes	Yes	Not applicable	Not applicable	Not applicable	Yes
Kale et al., 2013	Yes	no	No	Yes	Not applicable	Not applicable	Not applicable	Yes

Kantaputra et al., 2013	Yes	Yes	Yes	Yes	Not applicable	Not applicable	Not applicable	Yes
Kaul and Redd, 2008	Yes	Yes	Yes	Unclear	Not applicable	Not applicable	Not applicable	Yes
Kawamoto, Motohashi and Ohyama, 2004	Yes	Unclear	Yes	Yes	Not applicable	Not applicable	Not applicable	Yes
Khabour et al., 2010	Yes	Yes	Yes	Yes	Not applicable	Not applicable	Not applicable	Yes
Khurana et al., 2010	No	Unclear	Unclear	Yes	Not applicable	Not applicable	Not applicable	Yes
Kinyo et al., 2013	Yes	Yes	Yes	Yes	Not applicable	Not applicable	Not applicable	Yes
Kishore et al., 2014	Yes	Yes	Yes	Yes	Not applicable	Not applicable	Not applicable	Yes
Kobayashi et al., 2022	No	Yes	Yes	Yes	Not applicable	Not applicable	Not applicable	Yes
Kozma et al., 1999	Yes	Yes	Yes	Yes	Not applicable	Not applicable	Not applicable	Yes
Kramer et al., 2005	Yes	Yes	Yes	Yes	Not applicable	Not applicable	Not applicable	Yes
Kroigard et al., 2016	Yes	Yes	Yes	Yes	Not applicable	Not applicable	Not applicable	Yes
Levy et al., 2020	Yes	Yes	Yes	Yes	Not applicable	Not applicable	Not applicable	Yes
Liedén et al., 2016	Yes	Yes	Yes	Yes	Not applicable	Not applicable	Not applicable	Yes
Lin et al., 2022	No	Yes	Yes	Yes	Not applicable	Not applicable	Not applicable	Yes

Martinho et al., 2019	Yes	Yes	Yes	Yes	Not applicable	Not applicable	Not applicable	Yes
Marvin et al., 2011	Yes	Yes	Yes	Yes	Not applicable	Not applicable	Not applicable	Yes
Murata et al., 2019	Yes	Yes	Yes	Yes	Not applicable	Not applicable	Not applicable	Yes
O'Dwyer and Jones, 2005	Yes	no	No	no	Not applicable	Not applicable	Not applicable	Yes
Pipa Vallejo et al., 2008	Yes	Yes	Yes	Unclear	Not applicable	Not applicable	Not applicable	Yes
Reiche et al., 2014	Yes	Unclear	Yes	Yes	Not applicable	Not applicable	Not applicable	Yes
Retna and Sockalingnam, 2013	Yes	Unclear	Yes	Yes	Not applicable	Not applicable	Not applicable	Yes
Richieri-Costa et al., 1993	Yes	Yes	Yes	Yes	Not applicable	Not applicable	Not applicable	Yes
Rizos et al., 1998	Yes	Yes	Yes	Yes	Not applicable	Not applicable	Not applicable	Yes
Rock and McLellan, 2016	Yes	Yes	Yes	Yes	Not applicable	Not applicable	Not applicable	Yes
Shen et al., 2011	Yes	Yes	Yes	Yes	Not applicable	Not applicable	Not applicable	Yes
Siddiqui et al., 2021	No	Yes	Yes	Yes	Not applicable	Not applicable	Not applicable	Yes
Sikora et al., 2006	Yes	Yes	Yes	Unclear	Not applicable	Not applicable	Not applicable	Yes
Singh et al., 2017	Yes	Yes	Yes	Yes	Not applicable	Not applicable	Not applicable	Yes

Subramaniam and Neeraja, 2008	Yes	Yes	Yes	Yes	Not applicable	Not applicable	Not applicable	Yes
Suda et al., 2010	Yes	No	No	Yes	Not applicable	Not applicable	Not applicable	Yes
Sultan et al., 2020	Yes	Yes	Yes	Unclear	Not applicable	Not applicable	Not applicable	Yes
Sun et al., 2019	Yes	Yes	Yes	Yes	Not applicable	Not applicable	Not applicable	Yes
Talasila et al., 2017	No	Yes	Yes	Yes	Not applicable	Not applicable	Not applicable	Yes
Tanboga et al., 2001	Yes	Yes	Yes	Yes	Not applicable	Not applicable	Not applicable	Yes
Tanboga et al.,1992	Yes	Yes	Yes	Unclear	Not applicable	Not applicable	Not applicable	Yes
Tao et al., 2010	Yes	Yes	Yes	Yes	Not applicable	Not applicable	Not applicable	Yes
Tosun et al., 2006	Yes	Yes	Yes	No	Not applicable	Not applicable	Not applicable	Yes
Tuna et al., 2008/ Turkey	Yes	Yes	Yes	no	Not applicable	Not applicable	Not applicable	Yes
Tuna et al., 2009	Yes	Yes	Yes	Yes	Not applicable	Not applicable	Not applicable	Yes
Tuna et al., 2012	Yes	Yes	Yes	Yes	Not applicable	Not applicable	Not applicable	Yes
Vasudevan and Sinha, 2023	No	Yes	Yes	Yes	Not applicable	Not applicable	Not applicable	Yes
Waldron et al., 2010	Yes	Yes	Yes	no	Not applicable	Not applicable	Not applicable	Yes
Yin et al., 2013	Yes	Yes	Yes	Yes	Not applicable	Not applicable	Not applicable	Yes

Zidane and Alloussi, 2022	Yes	Yes	Yes	No	Not applicable	Not applicable	Not applicable	Yes

Supplementary Table 3. Biological processes characterized with the list of altered genes in syndromes with oligodontia.

#term ID	Term description	Observed gene count	Background gene count	False discovery rate	Matching proteins in your network
**GO:0042476**	Odontogenesis	7	126	1.56e-07	WNT10A,LEF1,AXIN2,EDA,MSX1,FGFR2,PITX2
**GO:0048732**	Gland development	8	419	7.29e-06	WNT10A,CDH1,LEF1,PTCH1,EDA,MSX1,FGFR2,PITX2
**GO:0060429**	Epithelium development	10	1069	1.65e-05	WNT10A,LEF1,AXIN2,NPHP1,PTCH1,EDA,DSP,MSX1,FGFR2,PITX2
**GO:0003007**	Heart morphogenesis	6	247	0.00015	AXIN2,PTCH1,DSP,MSX1,FGFR2,PITX2
**GO:0009887**	Animal organ morphogenesis	9	997	0.00015	WNT10A,LEF1,AXIN2,PTCH1,EDA,DSP,MSX1,FGFR2,PITX2
**GO:0003002**	Regionalization	6	341	0.00054	LEF1,AXIN2,PTCH1,MSX1,FGFR2,PITX2
**GO:0008544**	Epidermis development	6	320	0.00054	WNT10A,PTCH1,EDA,DSP,FGFR2,PITX2
**GO:0009653**	Anatomical structure morphogenesis	11	2229	0.00054	WNT10A,LEF1,AXIN2,NPHP1,PTCH1,EDA,DSP,MSX1,FGFR2,TBCE,PITX2
**GO:0030154**	Cell differentiation	13	3507	0.00054	WNT10A,CDH1,LEF1,AXIN2,NPHP1,PTCH1,EDARADD,EDA,DSP,MSX1,FGFR2,TBCE,PITX2
**GO:0048729**	Tissue morphogenesis	7	565	0.00054	WNT10A,LEF1,NPHP1,PTCH1,DSP,MSX1,FGFR2
**GO:0048762**	Mesenchymal cell differentiation	5	170	0.00054	WNT10A,LEF1,MSX1,FGFR2,PITX2
**GO:0060173**	Limb development	5	180	0.00054	LEF1,PTCH1,MSX1,FGFR2,PITX2
**GO:0030879**	Mammary gland development	4	128	0.0037	LEF1,PTCH1,MSX1,FGFR2

**GO:0061153**	Trachea gland development	2	2	0.0037	LEF1,EDA
**GO:0072359**	Circulatory system development	7	901	0.0040	LEF1,AXIN2,PTCH1,DSP,MSX1,FGFR2,PITX2
**GO:0007423**	Sensory organ development	6	577	0.0042	WNT10A,LEF1,NPHP1,MSX1,FGFR2,PITX2
**GO:0035108**	Limb morphogenesis	4	144	0.0044	LEF1,PTCH1,MSX1,FGFR2
**GO:0048731**	System development	12	3867	0.0046	WNT10A,CDH1,LEF1,AXIN2,NPHP1,PTCH1,EDA,DSP,MSX1,FGFR2,TBCE,PITX2
**GO:0071866**	Negative regulation of apoptotic process in bone marrow cell	2	4	0.0061	LEF1,FGFR2
**GO:0048513**	Animal organ development	11	3246	0.0062	WNT10A,CDH1,LEF1,AXIN2,NPHP1,PTCH1,EDA,DSP,MSX1,FGFR2,PITX2
**GO:0021873**	Forebrain neuroblast division	2	5	0.0077	LEF1,FGFR2
**GO:0007166**	Cell surface receptor signaling pathway	9	2040	0.0079	WNT10A,LEF1,AXIN2,PTCH1,EVC2,EDA,MSX1,FGFR2,IKBKG
**GO:0030900**	Forebrain development	5	391	0.0079	CDH1,LEF1,MSX1,FGFR2,PITX2
**GO:0023052**	Signaling	13	5057	0.0080	WNT10A,LEF1,AXIN2,NPHP1,PTCH1,EDARADD,EVC2,PCNT,EDA,DSP,MSX1,FGFR2,IKBKG
**GO:0048863**	Stem cell differentiation	4	183	0.0080	WNT10A,MSX1,FGFR2,PITX2
**GO:0008283**	Cell population proliferation	6	712	0.0085	LEF1,AXIN2,PTCH1,MSX1,FGFR2,PITX2
**GO:0071864**	Positive regulation of cell proliferation in bone marrow	2	6	0.0085	LEF1,FGFR2

**GO:0007154**	Cell communication	13	5165	0.0089	WNT10A,LEF1,AXIN2,NPHP1,PTCH1,EDARADD,EVC2,PCNT,EDA,DSP,MSX1,FGFR2,IKBKG
**GO:0061180**	Mammary gland epithelium development	3	61	0.0089	PTCH1,MSX1,FGFR2
**GO:0048699**	Generation of neurons	7	1131	0.0096	WNT10A,CDH1,LEF1,PTCH1,FGFR2,TBCE,PITX2
**GO:0010604**	Positive regulation of macromolecule metabolic process	11	3533	0.0099	WNT10A,CDH1,SRCAP,LEF1,AXIN2,PTCH1,EDA,MSX1,FGFR2,IKBKG,PITX2
**GO:0002009**	Morphogenesis of an epithelium	5	457	0.0117	WNT10A,LEF1,NPHP1,PTCH1,FGFR2
**GO:0007420**	Brain development	6	775	0.0117	CDH1,LEF1,PTCH1,MSX1,FGFR2,PITX2
**GO:0048568**	Embryonic organ development	5	454	0.0117	LEF1,PTCH1,MSX1,FGFR2,PITX2
**GO:0060348**	Bone development	4	216	0.0117	AXIN2,MSX1,FGFR2,PITX2
**GO:0045893**	Positive regulation of transcription, DNA-templated	8	1710	0.0129	CDH1,SRCAP,LEF1,PTCH1,MSX1,FGFR2,IKBKG,PITX2
**GO:0001837**	Epithelial to mesenchymal transition	3	79	0.0141	LEF1,MSX1,FGFR2
**GO:0001942**	Hair follicle development	3	80	0.0141	WNT10A,EDA,FGFR2
**GO:0061053**	Somite development	3	82	0.0149	LEF1,AXIN2,PTCH1
**GO:0042475**	Odontogenesis of dentin-containing tooth	3	83	0.0151	LEF1,EDA,MSX1

**GO:0050896**	Response to stimulus	15	7835	0.0152	WNT10A,CDH1,LEF1,AXIN2,NPHP1,PTCH1,EDARADD,EVC2,PCNT,EDA,DSP,MSX1,FGFR2,TBCE,IKBKG
**GO:0007165**	Signal transduction	12	4714	0.0162	WNT10A,LEF1,AXIN2,NPHP1,PTCH1,EDARADD,EVC2,PCNT,EDA,MSX1,FGFR2,IKBKG
**GO:0043588**	Skin development	4	266	0.0174	WNT10A,EDA,DSP,FGFR2
**GO:0016055**	Wnt signaling pathway	4	284	0.0206	WNT10A,LEF1,AXIN2,EDA
**GO:0048646**	Anatomical structure formation involved in morphogenesis	6	938	0.0206	LEF1,AXIN2,PTCH1,EDA,MSX1,FGFR2
**GO:0060070**	Canonical Wnt signaling pathway	3	100	0.0206	WNT10A,LEF1,EDA
**GO:0060349**	Bone morphogenesis	3	100	0.0206	AXIN2,MSX1,FGFR2
**GO:0022612**	Gland morphogenesis	3	104	0.0212	PTCH1,EDA,FGFR2
**GO:0071310**	Cellular response to organic substance	8	2019	0.0267	WNT10A,CDH1,LEF1,AXIN2,PTCH1,EDA,MSX1,FGFR2
**GO:0010092**	Specification of animal organ identity	2	18	0.0272	AXIN2,FGFR2
**GO:0042481**	Regulation of odontogenesis	2	18	0.0272	WNT10A,MSX1
**GO:0030111**	Regulation of Wnt signaling pathway	4	326	0.0289	LEF1,AXIN2,EDA,FGFR2
**GO:0030326**	Embryonic limb morphogenesis	3	120	0.0289	LEF1,PTCH1,MSX1
**GO:0043586**	Tongue development	2	20	0.0289	WNT10A,LEF1

**GO:0060537**	Muscle tissue development	4	323	0.0289	PTCH1,DSP,MSX1,FGFR2
**GO:0009790**	Embryo development	6	1049	0.0300	LEF1,AXIN2,PTCH1,MSX1,FGFR2,PITX2
**GO:0045667**	Regulation of osteoblast differentiation	3	125	0.0300	AXIN2,PTCH1,FGFR2
**GO:0030182**	Neuron differentiation	6	1062	0.0314	WNT10A,CDH1,PTCH1,FGFR2,TBCE,PITX2
**GO:0043009**	Chordate embryonic development	5	654	0.0314	LEF1,AXIN2,PTCH1,MSX1,FGFR2
**GO:0048518**	Positive regulation of biological process	13	6207	0.0314	WNT10A,CDH1,SRCAP,LEF1,AXIN2,NPHP1,PTCH1,PCNT,EDA,MSX1,FGFR2,IKBKG,PITX2
**GO:0010839**	Negative regulation of keratinocyte proliferation	2	22	0.0319	PTCH1,FGFR2
**GO:0060713**	Labyrinthine layer morphogenesis	2	22	0.0319	LEF1,FGFR2
**GO:0048754**	Branching morphogenesis of an epithelial tube	3	134	0.0340	LEF1,PTCH1,FGFR2
**GO:0007399**	Nervous system development	8	2188	0.0360	WNT10A,CDH1,LEF1,PTCH1,MSX1,FGFR2,TBCE,PITX2
**GO:0071495**	Cellular response to endogenous stimulus	6	1103	0.0360	WNT10A,CDH1,LEF1,AXIN2,MSX1,FGFR2
**GO:0030177**	Positive regulation of Wnt signaling pathway	3	141	0.0374	LEF1,EDA,FGFR2
**GO:0051716**	Cellular response to stimulus	13	6357	0.0374	WNT10A,CDH1,LEF1,AXIN2,NPHP1,PTCH1,EDARADD,EVC2,PCNT,EDA,MSX1,FGFR2,IKBKG

**GO:0000902**	Cell morphogenesis	5	708	0.0401	LEF1,PTCH1,MSX1,FGFR2,TBCE
**GO:0007435**	Salivary gland morphogenesis	2	27	0.0423	EDA,FGFR2
**GO:0010463**	Mesenchymal cell proliferation	2	27	0.0423	MSX1,FGFR2
**GO:0035148**	Tube formation	3	150	0.0426	PTCH1,EDA,FGFR2
**GO:0030030**	Cell projection organization	6	1158	0.0431	CDH1,NPHP1,PTCH1,PCNT,FGFR2,TBCE
**GO:0033688**	Regulation of osteoblast proliferation	2	28	0.0436	AXIN2,FGFR2
**GO:0001701**	In utero embryonic development	4	393	0.0450	LEF1,PTCH1,MSX1,FGFR2
**GO:0051252**	Regulation of RNA metabolic process	10	3759	0.0471	CDH1,SRCAP,LEF1,AXIN2,PTCH1,EDA,MSX1,FGFR2,IKBKG,PITX2
**GO:0048468**	Cell development	7	1719	0.0478	CDH1,LEF1,AXIN2,PTCH1,FGFR2,TBCE,PITX2
**GO:0031069**	Hair follicle morphogenesis	2	31	0.0496	WNT10A,FGFR2
**GO:0048522**	Positive regulation of cellular process	12	5584	0.0496	CDH1,SRCAP,LEF1,AXIN2,NPHP1,PTCH1,PCNT,EDA,MSX1,FGFR2,IKBKG,PITX2
**GO:0060325**	Face morphogenesis	2	31	0.0496	LEF1,MSX1
**GO:0060603**	Mammary gland duct morphogenesis	2	31	0.0496	PTCH1,FGFR2

Supplementary Table 4. Activated pathways characterized with syndromes with oligodontia.

#term ID	Term description	Observed gene count	Background gene count	False discovery rate	Matching proteins in your network
**hsa05200**	Pathways in cancer	7	515	4.55e-05	WNT10A,CDH1,LEF1,AXIN2,PTCH1,FGFR2,IKBKG
**hsa05217**	Basal cell carcinoma	4	63	4.55e-05	WNT10A,LEF1,AXIN2,PTCH1
**hsa05226**	Gastric cancer	5	146	4.55e-05	WNT10A,CDH1,LEF1,AXIN2,FGFR2
**hsa04390**	Hippo signaling pathway	4	154	0.00073	WNT10A,CDH1,LEF1,AXIN2
**hsa05213**	Endometrial cancer	3	58	0.0012	CDH1,LEF1,AXIN2
**hsa04064**	NF-kappa B signaling pathway	3	101	0.0046	EDARADD,EDA,IKBKG
**hsa05215**	Prostate cancer	3	97	0.0046	LEF1,FGFR2,IKBKG
**hsa04550**	Signaling pathways regulating pluripotency of stem cells	3	141	0.0101	WNT10A,AXIN2,FGFR2
**hsa05224**	Breast cancer	3	146	0.0101	WNT10A,LEF1,AXIN2
**hsa04310**	Wnt signaling pathway	3	154	0.0102	WNT10A,LEF1,AXIN2
**hsa04934**	Cushing syndrome	3	153	0.0102	WNT10A,LEF1,AXIN2
**hsa05225**	Hepatocellular carcinoma	3	161	0.0102	WNT10A,LEF1,AXIN2
**hsa05216**	Thyroid cancer	2	37	0.0132	CDH1,LEF1

**hsa04340**	Hedgehog signaling pathway	2	46	0.0185	PTCH1,EVC2
**hsa04520**	Adherens junction	2	69	0.0355	CDH1,LEF1
**hsa05221**	Acute myeloid leukemia	2	67	0.0355	LEF1,IKBKG
**hsa05412**	Arrhythmogenic right ventricular cardiomyopathy	2	77	0.0410	LEF1,DSP
**hsa05210**	Colorectal cancer	2	82	0.0437	LEF1,AXIN2
**hsa05165**	Human papillomavirus infection	3	324	0.0457	WNT10A,AXIN2,IKBKG
